# Change in C, N, and P Characteristics of *Hypericum kouytchense* Organs in Response to Altitude Gradients in Karst Regions of SW China

**DOI:** 10.3390/plants14152307

**Published:** 2025-07-26

**Authors:** Yage Li, Chunyan Zhao, Jiajun Wu, Suyan Ba, Shuo Liu, Panfeng Dai

**Affiliations:** College of Agriculture, Henan University of Science and Technology, Luoyang 471000, China; liyg18@lzu.edu.cn (Y.L.); zhaoocy@163.com (C.Z.); 18437919969@163.com (J.W.); basuyan0228@163.com (S.B.); m17838800978@163.com (S.L.)

**Keywords:** semi-evergreen shrub, ecological stoichiometric characteristics, altitudinal variation, plant organs, nutrient use strategy

## Abstract

The environmental heterogeneity caused by altitude can lead to trade-offs in nutrient utilization and allocation strategies among plant organs; however, there is still a lack of research on the nutrient variation in the “flower–leaf–branch–fine root–soil” systems of native shrubs along altitude gradients in China’s unique karst regions. Therefore, we analyzed the carbon (C), nitrogen (N), and phosphorus (P) contents and their ratios in flowers, leaves, branches, fine roots, and surface soil of *Hypericum kouytchense* shrubs across 2200–2700 m altitudinal range in southwestern China’s karst areas, where this species is widely distributed and grows well. The results show that *H. kouytchense* organs had higher N content than both global and Chinese plant averages. The order of C:N:P value across plant organs was branches > fine roots > flowers > leaves. Altitude significantly affected the nutrient dynamics in plant organs and soil. With increasing altitude, P content in plant organs exhibited a significant concave pattern, leading to unimodal trends in the C:P of plant organs, as well as the N:P of leaves and fine roots. Meanwhile, plant organs except branches displayed significant homeostasis coefficients in C:P and fine root P, indicating a shift in *H. kouytchense*’s P utilization strategy from acquisitive-type to conservative-type. Strong positive relationships between plant organs and soil P and available P revealed that P was the key driver of nutrient cycling in *H. kouytchense* shrubs, enhancing plant organ–soil coupling relationships. In conclusion, *H. kouytchense* demonstrates flexible adaptability, suggesting that future vegetation restoration and conservation management projects in karst ecosystems should consider the nutrient adaptation strategies of different species, paying particular attention to P utilization.

## 1. Introduction

Unique karst landforms are distributed across multiple regions worldwide, covering approximately 20% of the Earth’s terrestrial surface [1]. China has the largest area of karst landscapes, concentrated in its southwestern regions [1,2]. Due to the geological structure featuring exposed bedrock and scarce soil, coupled with a climate marked by distinct rainy and dry seasons, karst ecosystems face ecological challenges such as water scarcity, soil infertility, and declining biodiversity. These factors further exacerbate risks of vegetation degradation and soil erosion [3,4]. In this context, investigating plant communities, particularly highly adaptable shrub vegetation, in relation to environmental changes in karst regions from perspectives such as ecological stoichiometric characteristics and nutrient cycling is of great significance for ecological restoration and biodiversity conservation [5].

In recent years, ecological stoichiometric methods have been widely applied in plant ecology research, focusing on exploring the contents and ratios of carbon, nitrogen, phosphorus, and other elements in plant organs, as well as their variations across different ecosystems and scales [6,7,8]. These studies aim to reveal plant growth patterns, resource utilization strategies, and their interactions with environmental factors. For example, the ratio of nitrogen and phosphorus is commonly used to assess the nutrient limitation status in plant growth [9,10]; stoichiometric homeostasis reflects a plant’s stability when facing environmental fluctuations [11]; and plants also demonstrate stoichiometric flexibility to adapt to varying environmental conditions [12,13]. Therefore, applying ecological stoichiometry to analyze the nutrient status and stability of shrub vegetation in karst regions can provide a scientific basis for developing effective restoration strategies in ecologically fragile areas.

The ecological stoichiometric characteristics of plant communities in karst regions differ from those in other areas [14]. To better manage and protect karst regions, numerous scholars have conducted research on ecological stoichiometry in relation to factors such as degradation levels [15], vegetation types [16], ecological restoration measures [17], afforestation duration [18], and altitude [19]. These studies have revealed that these factors exert varying degrees of influence on plant or soil elements, thereby potentially altering the ecological functions and plant growth patterns in karst regions. For example, among different land-use types, forest soils exhibited significantly higher organic carbon and total nitrogen contents compared with shrub grassland, grassland, dry farmland, and paddy fields [16], suggesting that woody vegetation may be more conducive to nutrient accumulation and soil improvement. Through a study on the “leaves–litter–soil” system of plantations, Yu et al. found that the stand age had no significant effect on soil carbon, nitrogen, phosphorus, and their ratios, nor were there significant correlations with leaves or litter [18]. This indicates that plantation growth and material cycling may remain relatively stable within a certain time frame. Through studying the “leaves–fine root–litter–rhizosphere soil” system in natural forests, Hu et al. demonstrated that altitude significantly altered their carbon, nitrogen, phosphorus contents, and stoichiometric ratios, with significantly positive correlations observed among these components [19]. These findings highlight the crucial role of altitude in influencing plant growth and nutrient allocation among different plant organs. In summary, existing studies have primarily focused on variations in forest components such as leaves, litter, roots, and soil, while largely neglecting other plant organs like flowers and branches, particularly in natural shrub vegetation. There remains a notable lack of comprehensive analyses on the “flower–leaf–branch–root–soil” system in typical karst shrub ecosystems. Therefore, it is imperative to conduct systematic ecological stoichiometric research in natural shrub ecosystems.

This study focuses on *Hypericum kouytchense*, a species widely distributed in karst regions of Guizhou Province, southwestern China. We explored the carbon, nitrogen, and phosphorus stoichiometric characteristics of the “flower–leaf–branch–root–soil” system in *H. kouytchense* shrubs. We hypothesize that (1) the stoichiometric characteristics of flowers, leaves, branches, fine roots, and soil in *H. kouytchense* shrublands exhibit distinct altitudinal variation patterns; (2) there are certain coupling relationships between the stoichiometric characteristics of plant organs and soil; and (3) *H. kouytchense* demonstrates flexible adaptability to altitudinal changes and influences soil nutrient dynamics in karst environments. These findings will provide theoretical guidance for plant resource utilization and ecological restoration in karst regions and hold significant importance for a deeper understanding of the interactions between plant growth and environmental factors in ecologically fragile zones.

## 2. Results

### 2.1. Changes in C, N, and P Contents and Ratios

The C:N:P of different plant organs followed the order of branches > fine roots > flowers > leaves. All organs showed significantly higher C and N contents, C:P, and N:P compared with those of soil, while their C:N was significantly lower than that of soil (*p* < 0.05, Table 1). The C and N contents were highest in leaves, followed by flowers, with both being significantly greater than those in branches and fine roots (*p* < 0.05). P contents in branches and fine roots were significantly lower than those in flowers and leaves (*p* < 0.05), and flowers had the highest P contents (2.46 g·kg^−1^). The largest C:N and C:P existed in branches, which were significantly larger than those in fine roots, flowers, and leaves (*p* < 0.05), with the lowest C:N (5.46) in leaves and C:P (227.32) in flowers. The largest N:P (58.89) was observed in leaves, which was significantly larger than those in fine roots, branches, and flowers (*p* < 0.05), with the lowest N:P (33.59) being in flowers.

### 2.2. Altitudinal Patterns of C, N, and P Contents

**Figure 1:**
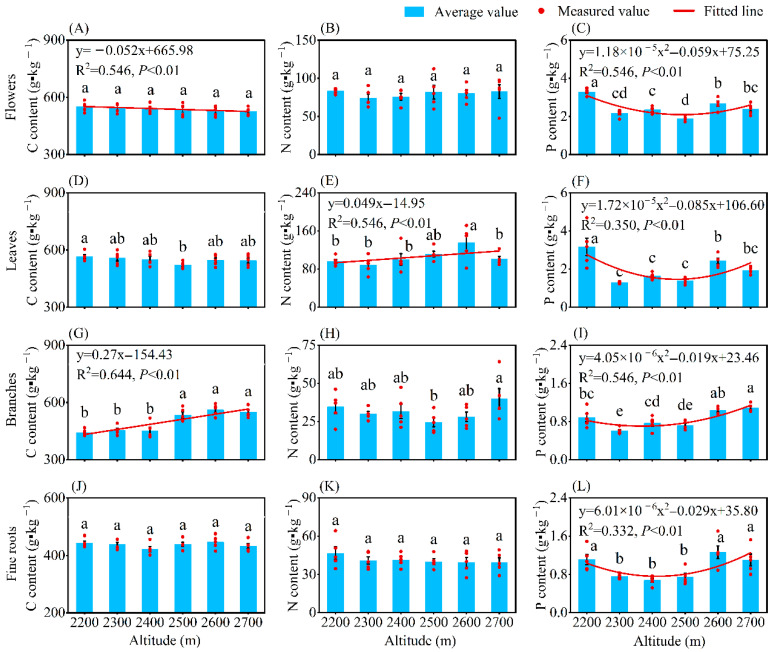
Changes in C, N, and P contents of plant organs along an altitudinal gradient. Note: (**A**) Flowers C content; (**B**) Flowers N content; (**C**) Flowers P content; (**D**) Leaves C content; (**E**) Leaves N content; (**F**) Leaves P content; (**G**) Branches C content; (**H**) Branches N content; (**I**) Branches P content; (**J**) Fine roots C content; (**K**) Fine roots N content; (**L**) Fine roots P content. Different lower-case letters indicate significant differences among altitudes (*p* < 0.05, Duncan’s test).

Altitude significantly affected the P content in plant organs, as well as the C and N contents in leaves and branches (*p* < 0.05), while the C and N contents in flowers and fine roots showed no significant changes (Figure 1). With increasing altitude, the C content of flowers decreased linearly and significantly (*p* < 0.01), whereas the C content of branches and the N content of leaves increased linearly and significantly (*p* < 0.01). The P content of plant organs all exhibited a significant concave pattern with increasing altitude (*p* < 0.01). The lowest P contents in leaves and branches were observed at an altitude of 2300 m, while the lowest P contents in flowers and fine roots appeared at an altitude of 2500 m and 2400 m.

**Figure 2:**
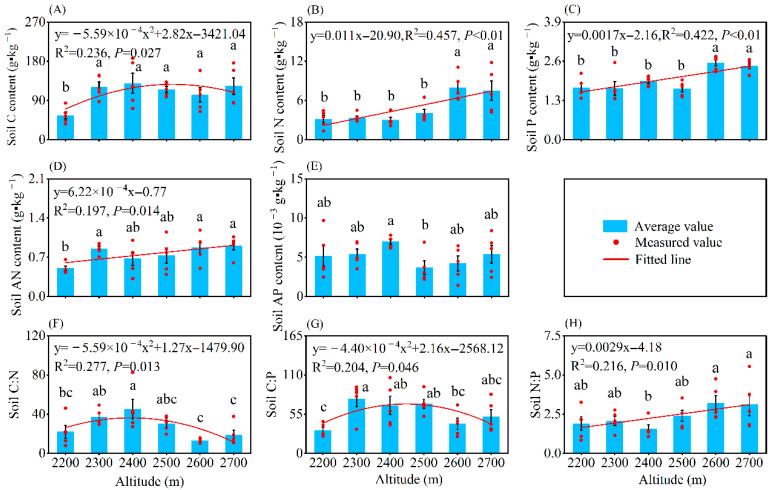
Changes in soil C, N, and P contents and their stoichiometric ratios along an altitudinal gradient. Note: (**A**) Soil C content; (**B**) Soil N content; (**C**) Soil P content; (**D**) Soil AN (available nitrogen) content; (**E**) Soil AP (available phosphorus) content; (**F**) Soil C:N; (**G**) Soil C:P; (**H**) Soil N:P. Different lower-case letters indicate significant differences among altitudes (*p* < 0.05, Duncan’s test).

Altitude also significantly altered soil elemental contents (*p* < 0.05, Figure 2A–E). Soil C content exhibited a unimodal pattern along the altitudinal gradient (*p* < 0.05), peaking at an altitude of 2400 m. However, soil N, P, and AN (available nitrogen) showed a linearly increasing pattern with rising altitude.

### 2.3. Altitudinal Patterns of C, N, and P Ratios

**Figure 3:**
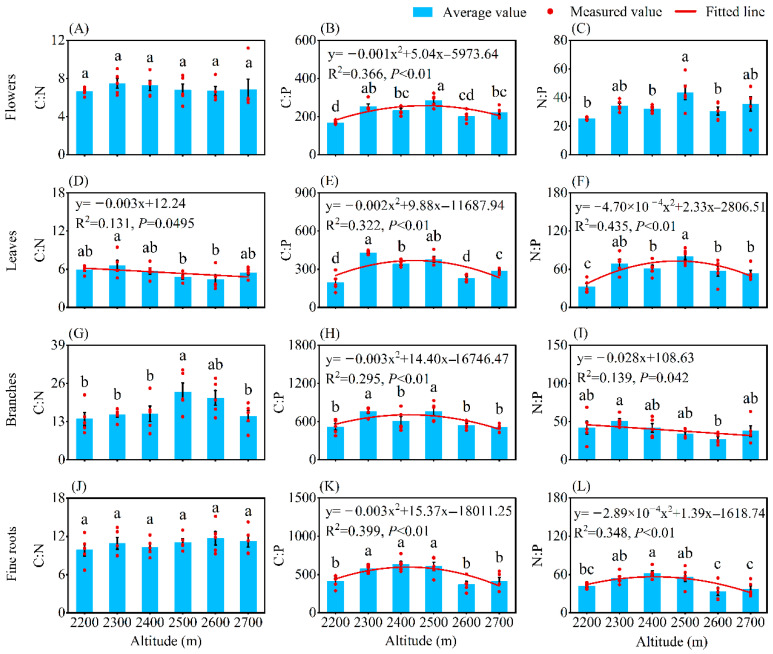
Changes in the stoichiometric ratios of plant organs along an altitudinal gradient. Note: (**A**) Flowers C:N; (**B**) Flowers C:P; (**C**) Flowers N:P; (**D**) Leaves C:N; (**E**) Leaves C:P; (**F**) Leaves N:P ; (**G**) Branches C:N; (**H**) Branches C:P; (**I**) Branches N:P; (**J**) Fine roots C:N content; (**K**) Fine roots C:P; (**L**) Fine roots N:P. Different lower-case letters express significant differences among altitudes (*p* < 0.05, Duncan’s test).

Except for the C:N of flowers and fine roots, there was significant variation in the element ratios of plant organs among different altitudes (*p* < 0.05, Figure 3). The leaf C:N and the branch N:P showed significant linear decreasing patterns with increasing altitude (*p* < 0.05). The C:P of all plant organs exhibited significant unimodal patterns along the altitudinal gradient (*p* < 0.01), with peak values occurring at 2500 m for flowers, 2300 m for leaves and branches, and 2400 m for fine roots. The altitudinal trend of N:P in leaves and fine roots was the same as that of C:P, peaking at 2500 m and 2400 m, respectively.

Soil element ratios were also significantly affected by altitude, with the soil N:P showing a significant linear increase with rising altitude (Figure 2F–H). However, both soil C:N and C:P first increased and then decreased with rising altitude (*p* < 0.05), peaking at 2400 m for soil C:N and 2300 m for soil C:P.

### 2.4. Ecological Stoichiometric Homeostasis

The P content of fine roots and C:P of flowers, leaves, and fine roots had different ecological stoichiometric homeostasis types (Table 2). The P content in fine roots demonstrated a weak plasticity (H = 1.98), which was between the plastic and weakly homeostatic types. However, the C:P of flowers showed the homeostatic (H = 4.14), while the C:P of leaves and fine roots displayed weak homeostasis (H = 2.53, 3.80). All other indicators of plant organs maintained the strictly homeostatic (∞, *p* > 0.05).

### 2.5. Correlation Relationships Among C, N, and P Contents and Ratios

**Figure 4:**
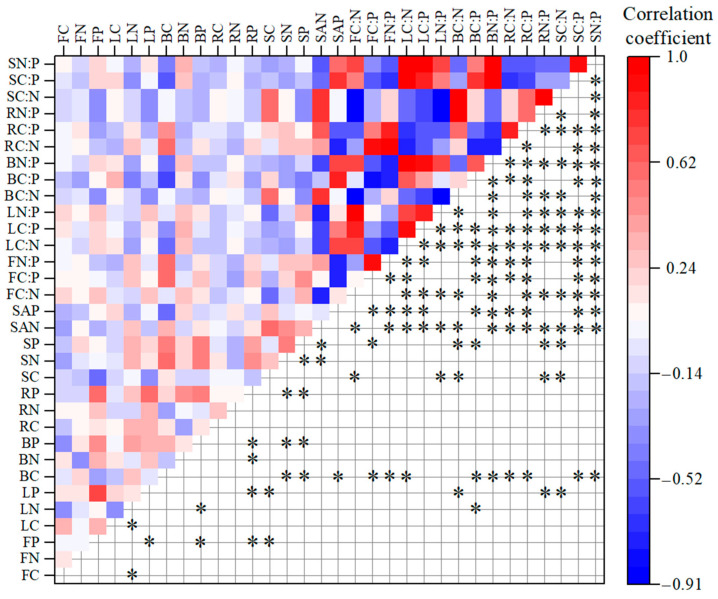
Correlations among C, N, and P contents and the stoichiometric ratios of plant organs and soil. Note: * indicates significant correlations (*p* < 0.05). FC, FN, FP, LC, LN, LP, BC, BN, BP, RC, RN, RP, SC, SN, SP, SAN, and SAP in Figure 4 denote carbon (C), nitrogen (N) and phosphorus (P) contents in flowers (F), leaves (L), branches (B), fine roots (R), and soil (S), as well as soil available nitrogen (AN) and available phosphorus (AP) contents. FC:N, FC:P, FN:P, LC:N, LC:P, LN:P, BC:N, BC:P, BN:P, RC:N, RC:P, RN:P, SC:N, SC:P, and SN:P in this figure represent the ratios of carbon, nitrogen, and phosphorus in flowers, leaves, branches, fine roots, and soil, respectively.

Pearson correlation heatmap illustrated the relationship among flowers, leaves, branches, fine roots, and soil (Figure 4). First, among elemental contents in different plant organs and soil, leaf N content showed significant negative correlations with C content in flowers and leaves, but leaf N content exhibited significant positive correlations with branch P content. A significant positive correlation was observed between branch N content and fine root P content. Significant positive correlations were found among P contents of all organs (flowers, leaves, branches, and fine roots), except for P contents between leaves and branches. Branch C and P contents, as well as fine root P content, were significantly positively correlated with soil N and P contents. Stoichiometric ratios of plant organs also showed significant correlations with soil AN and AP contents. Second, the stoichiometric ratios between plant organs and soil were more strongly correlated than their elemental contents. Specifically, flower C:P and N:P showed significant negative correlations with leaf C:N, branch C:P and N:P, and soil C:P and N:P. Conversely, flower C:P and N:P presented significant positive correlations with fine root C:N and C:P. Similarly, leaf C:N and C:P were significantly negatively correlated with branch C:N, soil C:N, and fine root C:N, C:P, and N:P. However, they showed significant positive correlations with branch C:P and N:P, and soil C:P and N:P. Branch C:P and N:P showed significant negative correlations with fine root C:N and C:P, but significant positive correlations with soil C:P and N:P. Notably, fine root C:P and N:P were significantly negatively correlated with soil N:P, while significantly positively correlated with soil C:N.

**Figure 5:**
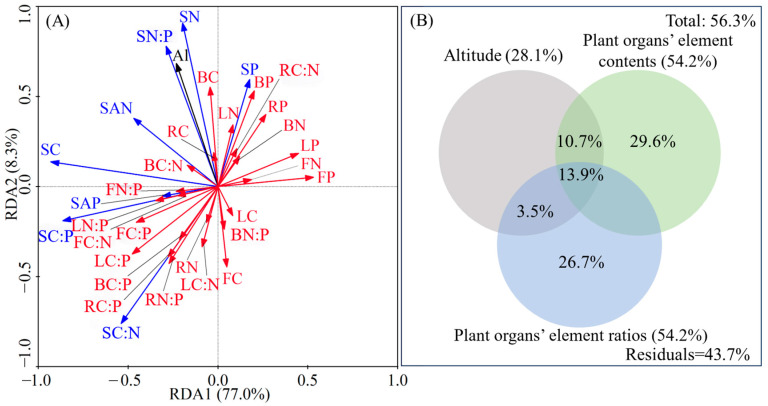
Relationships among altitude, plant elements and soil nutrients (**A**), and the contribution of altitude and plant elements to soil nutrient variability (**B**). The abbreviations in (**A**) maintain identical definitions to those provided in Figure 4.

To further elucidate the relationships among altitude, plant organs, and soil, RDA (redundancy analysis) and VPA (variance partitioning analysis) were conducted (Figure 5). The first and second axes of the RDA explained 85.3% of the total variation in all variables (Figure 5A). For plant organs, altitude had the greatest negative impact on flower C content but the greatest positive impact on branch C content. In soil, N content and N:P were also strongly positively affected by altitude. There were strong reciprocal relationships between the element contents of plant organs and soil N and P contents, as well as between stoichiometric ratios of plant organs and soil C:P and C:N. Overall, altitude, element contents of plant organs, and their ratios accounted for 56.3% of total nutrient variation. The independent contribution of plant organ element contents was the largest (29.6%), followed by plant organ element ratios (26.7%). The influence of altitude on soil nutrients was mediated by element contents and ratios of plant organs (Figure 5B).

## 3. Discussion

### 3.1. Variations in Element Contents and Stoichiometric Ratios of H. kouytchense Shrublands in Karst Regions

C, N, and P are essential macronutrients for plant growth and development, and their deficiency or imbalance can directly impact the structure and stability of ecosystems [12,20,21]. To better protect the fragile karst habitats, this study investigated the variations in C, N, and P contents and their ratios in a typical natural shrub community (*H. kouytchense*) in the karst region, which was crucial for ecological restoration. The C content in different plant organs ranged from 437.35 g·kg^−1^ to 547.82 g·kg^−1^, which was basically consistent with the global average for woody plants (476 g·kg^−1^) [22]. Surprisingly, the N content (31.49–105.55 g·kg^−1^) in these plant organs was relatively high, particularly in leaves, where it significantly exceeded the leaf N levels of global (18.4 g·kg^−1^) and Chinese terrestrial plants (18.6 g·kg^−1^) [23,24], as well as those in plantations (46 g·kg^−1^) in karst areas [25]. We identified the possible reasons for this result as follows: (1) The soil N content (4.82 g·kg^−1^) in this study was higher than that in other studies (2.30 g·kg^−1^, 2–3.5 g·kg^−1^) [19,26], providing sufficient N sources for plant uptake, which was conducive to nitrogen accumulation in plants. (2) A comprehensive study showed that the leaf N content of 68 plant species in karst regions varied from 5.01 g·kg^−1^ to 41.62 g·kg^−1^ [27], indicating significant variability in N content among plants in this region, with species-specific differences. (3) In the harsh karst habitat, *H. kouytchense* was widely distributed and grew well, suggesting that it may have the ability to enrich nutrients, particularly N, to enhance its adaptability to adverse environmental conditions. The P content in all plant organs ranged from 0.85 g·kg^−1^ to 2.46 g·kg^−1^, with the level of fine root P content (0.95 g·kg^−1^) being essentially equal to that of global terrestrial plants (0.94 g·kg^−1^) [28]. However, the leaf P content (1.97 g·kg^−1^) was higher than that of global terrestrial plant leaves (1.43 g·kg^−1^) [24], but lower than that of plantations in karst regions (about 2.5 g·kg^−1^) [25]. This suggests that plant P content in karst areas was generally higher, and such differences may be related to variations in vegetation types and their habitats. In published studies, leaves and fine roots have received extensive attention, while organs such as flowers and branches have been less investigated. The only existing ecological stoichiometry study on *Ammopiptanthus mongolicus* that included floral organs revealed that flower C content was lower than that of leaves and roots, whereas flower N and P contents were higher than those of leaves and roots [29]. In this study, flower C and N contents were lower than in leaves but higher than in roots, while flower P content was higher than in both leaves and fine roots (Table 1). This indicates that pants generally allocated more N and P to reproductive organs (such as flowers) during reproductive growth, while also strategically distributing C to maintain vegetative growth in stressful habitats. The higher N content in leaves compared with flowers may illustrate that the plant was in a state of N accumulation, where the N demand of flowers had already been met, resulting in excess N being stored in leaves. Overall, the C, N, and P contents in both flowers and leaves were significantly higher than those in branches and fine roots (Table 1). Jing et al. analyzed the stoichiometric traits of six plantations and found that leaf C, N, and P contents were generally higher than those of roots in all tree species, while branch C, N, and P contents showed no consistent pattern compared with leaves or roots [30]. This certified that plants allocated absorbed elements in similar proportions between leaves and roots, whereas branch elements exhibited greater variability and were more strongly influenced by species. Therefore, *H. kouytchense* shrublands in the karst region exhibit a higher N content, while maintaining normal levels of C and P contents. Its flowers and leaves have significantly higher C, N, and P contents than both branches and fine roots, which directly influences their C:N:P stoichiometric ratios.

In this study, the flower C:N, C:P, and N:P of *H. kouytchense* was 6.96, 227.32, and 33.59, respectively (Table 1), which was smaller than the C:N but larger than the C:P and N:P in *A. mongolicus* flowers [29]. This discrepancy arose because flowers of *H. kouytchense* possessed higher N content but lower P content than *A. mongolicus*. Globally, the leaf N:P for terrestrial plants ranges between 10.20 and 35.40 [31], while the leaf N:P for Chinese terrestrial plants is 14.4 [23], both significantly lower than our finding (58.89). This exceptionally high N:P in *H. kouytchense* leaves may be attributed to the following two key factors: (1) the inherently higher leaf N content in this species, and (2) the potential P limitation phenomenon in karst ecosystems [19,25]. This adaptive response aligns with the existing evidence, which demonstrated that an increased leaf N content plays a vital role in mitigating phosphorus stress [32]. Furthermore, the C:N and C:P of flowers, leaves, and fine roots were significantly lower than those of branches, due to the minimal N and P contents in branches. However, the N:P of branches was markedly lower than that of leaves and fine roots, attributable to the significantly lower N content in branches (31.49 g·kg^−1^) compared with both leaves (105.55 g·kg^−1^) and fine roots (41.42 g·kg^−1^) (Table 1). Our results show that the observed C:N:P ratios for leaves, branches, and fine roots differed from those reported in both Chinese forest [6] and karst plantations [25], demonstrating that species-specific stoichiometric signatures overrode methodological influences to some extent. In terms of different organs, branches and fine roots exhibited higher C:N:P ratios than leaves, which was supported by the reported results of Zhang et al. [6] and Zhang et al. [33]. This indicated that despite differences in vegetation type (species), research scale, and statistical methods, the variations in stoichiometric ratios among organs remained relatively stable. Specifically for leaves versus fine roots, leaves showed significantly lower C:N and C:P but higher N:P ratios compared with fine roots. These findings aligned with the results reported by Hu et al. [19] and Xiong et al. [34]. The consistent patterns across studies further confirm the stability of inter-organ differences in stoichiometry ratios, revealing that different species may follow similar nutrient utilization and allocation strategies.

At the global level, the soil C content at 0.5–100 cm depth was 41.3 g·kg^−1^ on average, with a maximum value of 545 g·kg^−1^ [35]; soil N content at 0–35 cm depth ranged from 1 g·kg^−1^ to 4 g·kg^−1^ [36]; and soil P content at 0–30 cm depth was 0.597 g·kg^−1^, with a range of 0.003–9.63 g·kg^−1^ [35]. At the level of karst regions, surface soil C, N, and P contents were 45.61, 2.54, and 0.97 g·kg^−1^ [15]. Compared with these global and regional levels, *H. kouytchense* shrublands exhibited clearly higher soil nutrient concentrations, with averages of 108.07 g·kg^−1^ (C), 4.82 g·kg^−1^ (N), and 2.01 g·kg^−1^ (P). The soil C:N:P ratio (57:5:1) in our study exceeded that of two plantations in karst regions [25] but remained lower than that of Chinese forests [6]. These differences may stem from both the higher soil C content in *H. kouytchense* shrublands and inconsistent data calculation methods in Zhang et al. [6]. In addition, the higher soil C and P contents led to a greater soil C:N but smaller C:P and N:P compared with the findings of Wang et al. [15]. At the same time, the enriched soil N and P pools likely enhanced N and P availability, as evidenced by the higher soil AN and AP contents (Figure 2), compared with those reported for karst plantations [26]. Consequently, *H. kouytchense* shrublands maintain abundant soil C, N, and P reserves. If no soil erosion occurs in the future, these nutrient pools could provide sustainable supplies for long-term plant growth.

### 3.2. Altitudinal Effects on Element Contents and Stoichiometric Ratios of H. kouytchense Shrublands

As is well known, altitude, which affects moisture and temperature, is a critical factor influencing vegetation growth and nutrient cycling [37,38]. Within the altitude range of 2200–2700 m, significant variations were observed in the P content of flowers and fine roots, as well as the C, N, and P contents of leaves, branches, and soil (Figure 1 and Figure 2). This indicates that altitude played an important role in the physiological metabolic activities and nutrient allocation across different plant organs and soil, as well as reflecting the adaptive adjustments of *H. kouytchense* to altitudinal gradients. With increasing altitude, the P content in plant organs first decreased significantly and then increased, while soil P content showed a significant linear increase, which was consistent with the results of Hu et al. [19]. Similarly, Li et al. reported a significant increase in soil P content between altitudes of 2600 and 3200 m [37]. In low-altitude areas, plants grew rapidly and had a high demand for P. To adapt to P limitation, plants reduced their P requirements, leading to lower P content in plant organs. Concurrently, soil leaching contributed to P loss and downward migration, resulting in lower soil P content. In contrast, at higher altitudes, enhanced physical weathering released P from rocks, promoting the accumulation of P in soils. To cope with strong winds and low temperatures, plants reduced their height and grew more slowly, leading to P accumulation in their organs. This revealed a shift in the P utilization strategy of *H. kouytchense* from “competitive acquisition” to “conservative storage” along the altitudinal gradient. We observed a significant increase in leaf N content with increasing altitude, likely due to enhanced N investment in leaves to maintain photosynthetic efficiency and leaf protection. As shown in Table 1, leaf N content was significantly higher than that of other organs. Soil N and AN contents also increased markedly with altitude, attributed to slower plant growth rates and litter decomposition, leading to N accumulation. Wang et al. reported similar trends in soil AN content, but no significant trends were found in the N content in leaves and fine roots [39], possibly due to different species and altitude gradients. In contrast to N and P, the C dynamics in plant organs and soils exhibited more complex variations along the altitudinal gradient. Flower C content decreased significantly, while branch C content increased significantly. Soil C content showed a unimodal change trend. From low to high altitudes, environmental stresses (e.g., low temperatures, strong winds, and intense radiation) likely drove plants to prioritize C allocation to branches for growth and defense, reducing reproductive investment. Similar C allocation strategies had been reported in *A. mongolicus* from saline–alkali habitats, where stems and leaves had higher C content than flowers and seeds, but this pattern was not found in other sandy habitats [29]. This suggested similar C allocation strategies among species under harsh conditions. The unimodal trend in soil C content likely reflected a balance between litter formation and decomposition at mid-altitude areas, while reduced soil C supplement at high-altitude areas resulted from both decreased litter input and slower decomposition rates. Our results demonstrate asynchronous allocation of C, N, and P in *H. kouytchense*, highlighting its efficient resource-allocating strategies and flexible adaptability across altitudinal gradients.

Except for the flower C:N, the stoichiometric ratios of flowers, leaves, branches, and fine roots showed significant variations across altitudinal gradients. Specifically, their C:P, as well as the N:P of leaves and fine roots, initially increased and then decreased significantly with rising altitude, in contrast to the variation in P content in plant organs. Along the altitudinal gradient, the leaf C:N decreased significantly, in contrast to the increase in leaf N content (Figure 1 and Figure 3). However, Qin et al. found different patterns in *Picea crassifolia*, where leaf C:P and N:P increased significantly with rising altitude, while the C:P and N:P in branches and fine roots first decreased and then increased [38]. Similarly, Bin et al. pointed that leaf C:N and C:P decreased significantly with rising altitude [40]. Although these studies examined different altitude ranges, locations, and plant species, they consistently demonstrated that changes in elemental ratios were inversely correlated with variations in the denominator elements (N or P). This indicates that the altitudinal patterns of C:P and N:P were primarily driven by changes in P content, and C:N variations were determined by dynamics of the N content. This phenomenon occurred because N and P exhibited higher metabolic sensitivity than C under stressful environmental conditions, reflecting their crucial roles in plant physiological strategies and elemental functionality. Branch N:P decreased significantly, and this seemed to be unrelated to changes in branch N and P content. This is perhaps because the growth of *H. kouytchense* was not limited by N or P, resulting in their allocation within branches being independent of each other. In the study by Qin et al. [38], branch N:P initially decreased and then increased with altitude, exhibiting an opposite trend to branch P content. This was attributed to the leaf N:P of *P. crassifolia* being <14, suggesting potential N limitation, which caused branch N:P variations to depend on P content. In this study, the N:P in leaves exceeded 16 at any altitude, which would typically indicate P limitation in its growth [26,41]. However, since the leaf P content and soil P and AP contents of *P. crassifolia* shrublands were higher than in other studies [19,24,37], we concluded that the elevated leaf N:P might instead have resulted from an excessively high leaf N content (Table 1). Soil C:N and C:P initially increased and then decreased significantly with rising altitude, primarily influenced by changes in soil C content. In contrast, the soil N:P increased linearly, consistent with variations in soil N and P contents. This pattern aligned with the findings of Qin et al. [38]. However, Bin et al. reported that soil C:P and N:P increased significantly with rising altitude, corresponding to changes in soil C, N, and P contents [40]. On the one hand, this suggested that the relationship between elemental ratios and elemental contents in soil was more complex than in plant organs, possibly determined by the relative changes in different elements rather than the absolute variation in a single element. On the other hand, it implied that changes in soil elemental ratios and contents resulted from the combined effects of plants and altitude (Figure 5B) [42].

A comparison of elemental contents and ratios among plant organs and soil found that only the flowers, leaves, branches, fine roots, and soil exhibited completely consistent trends in C:P variation along the altitudinal gradient, which was inversely related to P changes in plant organs (Figure 2 and Figure 3). Furthermore, in this study, only the C:P of flowers, leaves, and fine roots, as well as the P content in fine roots, displayed different homeostasis coefficients, while other plant organs maintained strict homeostasis for C, N, and P contents and their ratios (Table 2). We speculate that it may be related to plant P uptake and utilization strategies. Hu et al. found that leaf P re-absorption efficiency initially decreased and then increased significantly with rising altitude [19], suggesting a shift in plant P utilization from an exploitative to a conservative strategy. Overall, these results support our Hypotheses 1 and 3. The C, N, and P contents and their ratios in *H. kouytchense* plant organs and soil exhibit distinct altitudinal variation patterns; the species demonstrates flexible environmental adaptability, reflecting its flexible environmental adaptability.

### 3.3. Relationship Between Plant Organs and Soil

With the exception of a non-significant positive correlation between P content in leaves and branches, significant positive correlations were observed in P content among different plant organs, indicating strong and actively coupled relationships in P allocation across organs. Except for the non-significant positive correlations in leaf P, flower P with soil C, the C, N, and P of plant organs showed significant positive correlations with soil nutrients (Figure 4). Jia et al. conducted a P-addition experiment in *Pinus elliottii* plantations and found significant positive correlations in C, N, and P contents among leaves, branches, fine roots, litter, and soil [43]. This demonstrated that under P-rich soil conditions, positive correlations emerged both among plant organs and between plant organs and soil. Moreover, significant correlations also existed between elemental ratios in plant organs and soil. This occurred because N and P were primarily absorbed by fine roots from the soil, then transported upwards and allocated. Simultaneously, leaves, as the primary photosynthetic organs, maintained a nutrient surplus that could be redistributed to other organs. And leaf litter formed a critical linkage with soil, which was decomposed to supplement soil nutrients and re-enter the plant through roots [44]. Therefore, in *H. kouytchense* shrublands, P drives coordinated changes in both nutrients and stoichiometric ratios among leaves, flowers, branches, fine roots, and soil. This results in strong coupling relationships across these organs, thereby supporting Hypothesis 2.

As a whole, the RDA revealed that the first two axes collectively explained 85.3% of the total variation in all indicators among altitude, plant organs, and soil (Figure 5A). Altitude and plant organs accounted for 56.3% of the contribution to soil nutrient stoichiometric characteristics, with altitude effects being mediated through plant organs (Figure 5B). These results demonstrate that our dataset effectively elucidated the complex and close interrelationships among them, consistent with previous findings on strong altitude–soil–plant organ interactions [19,38]. In the RDA of Figure 5A, plant organ element contents clustered with soil P in the first quadrant, while plant organ element ratios co-occurred with soil AP in the third quadrant, indicating positive correlations between these variables. This is similar to the correlation analysis results shown in Figure 4, confirming P as the key driver of nutrient cycling in *H. kouytchense* shrublands. These findings corroborate Hu et al.’s results in karst ecosystems, where P was identified as the critical factor governing nutrient cycling in *Broussonetia papyrifera* forests [19].

## 4. Materials and Methods

### 4.1. Research Area

This study was conducted in the Jiucaiping Nature Reserve (26.35–27.46° N, 104.41–105.30° E; approximate altitude range: 2100–2780 m a.s.l.), located in Hezhang County, Bijie City, Guizhou Province, southwestern China. This area is characterized by a warm-temperate cool climate, with a mean annual temperature of 10.1 °C and a mean annual precipitation of 1028.4 mm (meteorological data source: Bijie Meteorological Bureau). Based on the international soil classification system (WRB) and our field observations, the dominant soil types are roughly Acrisols, Luvisols, and Gypsisols. The natural vegetation is primarily alpine meadows and shrublands, where species of *Rhododendron*, *Spiraea*, and *Salix* occur sparsely or in localized patches, while *H. kouytchense* exhibits the most extensive distribution.

### 4.2. Experimental Design and Sample Collection

Along the altitudinal gradient, we set up a single transect with six sampling sites at 2200, 2300, 2400, 2500, 2600, and 2700 m a.s.l., respectively, all maintaining similar slope aspects. Five 5 m × 5 m quadrats at each site were randomly established to measure the plant height and crown width of *H. kouytchense*. Detailed information is shown in Table 3.

In each quadrat, five *H. kouytchense* individuals were randomly selected to collect plant samples, including their flowers, leaves, branches, and fine roots (<2 mm), and each organ type was fully mixed into one plant sample. Then, according to the five-point sampling method, five samples of surface soil (0–20 cm) per quadrat were collected and mixed into one soil sample. A total of 30 flowers, 30 leaves, 30 branches, 30 fine roots, and 30 soil samples were obtained. All samples were naturally air-dried to a constant weight. Plant samples were crushed and stored in polyethylene ziplock bags, while soil samples were sieved through a 2 mm mesh before bagging. These processed samples were preserved for subsequent chemical element analysis.

### 4.3. Chemical Element Determination

The carbon and nitrogen contents of plant samples (flowers, leaves, branches, and fine roots) were determined using the combustion method [45], and the phosphorus content in plant samples was quantified via the molybdenum–antimony colorimetric method [46].

Soil organic carbon content was measured via the potassium dichromate titration method with concentrated sulfuric acid [47], while soil total nitrogen content was determined using the combustion method [45], and soil total phosphorus content was quantified via the molybdenum–antimony colorimetric method [46]. The alkali–hydrolysis diffusion method was used to determine soil available nitrogen (AN) content [48], and soil available phosphorus (AP) content was measured using the molybdenum–antimony colorimetric method after extraction with sodium bicarbonate [48].

### 4.4. Statistical Analysis

In this study, soil carbon, nitrogen, and phosphorus referred specifically to soil organic carbon, total nitrogen, and total phosphorus, respectively. The carbon (C), nitrogen (N), phosphorus (P) contents and C:N, C:P, and N:P of plant organs and soil, as well as soil AN and AP contents, were presented using the mean value with standard error. A normality test was conducted using SPSS 22.0 (International Business Machines Corporation, Chicago, IL, USA), and all datasets were confirmed to follow normal distribution. One-way ANOVA and Duncan’s multiple range test were performed using SPSS 22.0 to compare the differences among different plant organs and soil, and across altitudinal gradients, with statistical significance set at *p* < 0.05. Different regression analyses in Origin 2021 (OriginLab Corporation, Northampton, MA, USA) were used to linearly fit the contents and ratios of all elements, exploring their variation patterns along the altitudinal gradient. Following the method of Sterner and Elser [49], we calculated the stoichiometric homeostasis coefficients (H) of different plant organs using the formula “y = c·x^1/H^” in Excel 2017 (Microsoft Corporation, Redmond, WA, USA). In this formula, y represents the elemental contents (C, N, and P) or their ratios in plant organs, x denotes the corresponding soil nutrient parameters (C, N, P, AN, AP, C:N, C:P, and N:P), c represents the constant, and the H denotes the homeostasis coefficient. According to the criteria established by Makino [50] and Persson [51], H was classified into six distinct categories: no homeostatic (H = 1), plastic (1 < H <$\;1.\dot{3}$, *p* < 0.05), weakly plastic 1 < H < 2, *p* < 0.05), weakly homeostatic (2 < H < 4, *p* < 0.05), homeostatic (H > 4, *p* < 0.05), and strictly homeostatic (∞, *p* > 0.05). These categories were used to evaluate the capacity of plant organs to maintain internal elemental balance under environmental variations. Pearson correlation analysis, redundancy analysis (RDA), and variance partitioning analysis (VPA) were conducted to elucidate the relationships among altitude, stoichiometric characteristics of plant organs, and soil properties, using Origin 2021, Canoco 4.5 (Biometris, Wageningen, The Netherlands), and R version 4.4.0 Vegan package (R Foundation for Statistical Computing, Vienna, Austria), respectively.

## 5. Conclusions

Our study investigated the stoichiometric variations in C, N, and P in the integrated “flower–leaf–branch–fine root–soil” system of *H. kouytchense* shrublands along an altitudinal gradient (2200–2700 m). The C and P contents in plant organs and soils of *H. kouytchense* shrublands in karst regions of China fall within the normal ranges presented in published studies, whereas their N contents are notably higher. The altitudinal variation patterns of C, N, and P contents and stoichiometric ratios across different plant organs and soil exhibit considerable complexity, with increasing altitude, significant linear decreases exist in flower C content, leaf C:N, and branch N:P. Conversely, significant linear increases occur in branch C content, leaf N content, and soil N content, P content, AN content, and N:P. Notably, P contents in flowers, leaves, branches, and fine roots show a significant unimodal altitudinal pattern (first decreasing and then increasing), while their N:P significantly increases first and then decreases. This concave altitudinal pattern is also found in leaf N:P, fine root N:P, and soil C:N and C:P, along with soil C content. Tight nutrient coupling exists among these plant organs and soil, primarily driven by P as the key regulator. Moreover, *H. kouytchense* demonstrates remarkable adaptive flexibility to both soil nutrients and altitudinal changes. As altitude increases, it shifts its P utilization strategy from acquisitive to conservative. Therefore, future vegetation restoration and management practices in karst regions should prioritize the P adaptation strategies of plant species.

## Figures and Tables

**Table 1:** C (carbon), N (nitrogen), and P (phosphorus) contents and the stoichiometric ratios of plant organs and soil.

Indicators	Flowers	Leaves	Branches	Fine roots	Soil
C (g·kg^−1^)	539.14 ± 4.5 a	547.82 ± 5.31 a	498.77 ± 10.54 b	437.35 ± 3.41 c	108.07 ± 7.31 d
N (g·kg^−1^)	79.74 ± 2.41 b	105.55 ± 4.48 a	31.49 ± 1.8 d	41.42 ± 1.37 c	4.82 ± 0.49 e
P (g·kg^−1^)	2.46 ± 0.09 a	1.97 ± 0.14 b	0.85 ± 0.04 c	0.95 ± 0.05 c	2.01 ± 0.08 b
C:N	6.96 ± 0.24 cd	5.46 ± 0.24 d	17.35 ± 1.07 b	10.87 ± 0.34 c	27.74 ± 2.93 a
C:P	227.32 ± 8.2 d	308.91 ± 16.61 c	618.82 ± 26.9 a	501.98 ± 25.02 b	56.5 ± 4.52 e
N:P	33.59 ± 1.58 c	58.89 ± 3.5 a	38.85 ± 2.42 c	47.42 ± 2.66 b	2.38 ± 0.2 d
C:N:P	219:80:1	277:106:1	589:31:1	462:41:1	57:5:1

Note: different lower-case letters in each line represent significant differences in element contents or their stoichiometric ratios among flowers, leaves, branches, fine roots, and soil (*p* < 0.05, Duncan’s test).

**Table 2:** Homeostasis coefficients of plant organs.

Organ	Indicator	C	N	P	AN	AP	C:N	C:P	N:P
Flowers	H	∞	∞	∞	∞	∞	∞	4.14	∞
	*p*	>0.05	>0.05	>0.05	>0.05	>0.05	>0.05	<0.05	<0.05
Leaves	H	∞	∞	∞	∞	∞	∞	2.53	∞
	*p*	>0.05	>0.05	>0.05	>0.05	>0.05	>0.05	<0.05	<0.05
Branches	H	∞	∞	∞	∞	∞	∞	∞	∞
	*p*	>0.05	>0.05	>0.05	>0.05	>0.05	>0.05	>0.05	>0.05
Fine root	H	∞	∞	1.98	∞	∞	∞	3.80	∞
	*p*	>0.05	>0.05	<0.05	>0.05	>0.05	>0.05	<0.05	>0.05

Note: Homeostasis coefficients (H) were calculated from the C, N, and P contents of plant organs under soil C, N, P, AN, and AP conditions, as well as the C:N, C:P, and N:P of plant organs under soil C:N, C:P, and N:P conditions. The significance level was *p* < 0.05.

**Table 3:** Sampling site information and growth profile of *H. kouytchense*.

Sample Site No.	Altitude (m)	Latitude (°N)	Longitude (°E)	Average Plant Height (cm)	Average Crown Width (cm)
1	2200	26.9534	104.7572	97.4 ± 5.2	87.3 ± 26.0
2	2300	26.9866	104.7623	90.0 ± 7.0	65.7 ± 16.4
3	2400	26.9883	104.7599	66.0 ± 3.1	55.7 ± 11.9
4	2500	26.9903	104.7578	53.0 ± 3.8	73.3 ± 17.2
5	2600	26.9911	104.7557	54.4 ± 7.4	62.0 ± 15.5
6	2700	26.9923	104.7537	38.0 ± 1.8	64.5 ± 20.0

## Data Availability

All data are presented in the main text.
